# Supplementary material for: Probiotic Potential of a Folate-Producing Strain Latilactobacillus sakei LZ217 and Its Modulation Effects on Human Gut Microbiota
Source: Foods. 2022 Jan 16;11(2):234. doi: 10.3390/foods11020234 (PMC8774781; doi:10.3390/foods11020234)
Supplement: Supplementary file 1 [file foods-11-00234-s001.zip › foods-1526213-supplementary.pdf]

Supplementary Material

Figure Captions

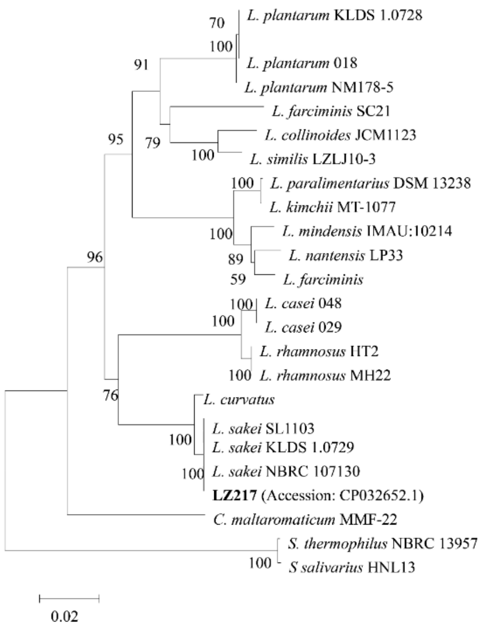

Figure S1. The phylogenetic tree of *Lactobacillus sakei* LZ217 based on 16S rDNA sequence.

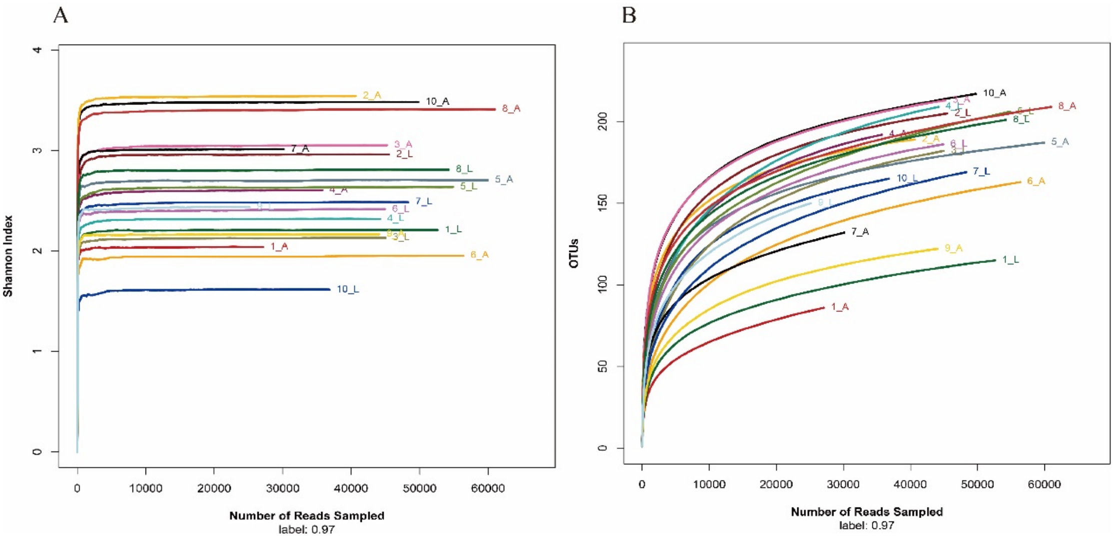

Figure S2. Shannon curves and rarefaction curves. (A) Shannon index of sample sequence. (B) The rarefaction curve obtained from the sample sequencing. Note: 1-10 indicates volunteer number, "_ A" for the VIS group, "_ L" for VIL group.

Tables

Table S1. Screening of folate producers from raw cow milk.

Strain	Growth in Folic Acid Assay Broth	Extracellular folate (ng/mL)	Intracellular folate (ng/mL)	Total folate (ng/mL)
LZ54	-	ND	ND	ND
LZ55	-	ND	ND	ND
LZ201	-	ND	ND	ND
LZ202	-	ND	ND	ND
LZ216	-	ND	ND	ND
LZ225	-	ND	ND	ND
LZ231	-	ND	ND	ND
LZ204	-	ND	ND	ND
LZ217	+	37.88 ± 0.02	201.82 ± 0.06	239.70 ± 0.03
LZ220	+	4.20 ± 0.05	1.30 ± 0.05	5.50 ± 0.02
LZ221	-	ND	ND	ND
LZ223	-	ND	ND	ND
LZ229	-	ND	ND	ND
LZ232	-	ND	ND	ND
LZ206	+	19.44 ± 0.01	42.62 ± 0.05	62.06 ± 0.03
LZ227	-	ND	ND	ND

LZ228	-	ND	ND	ND
LZ4	+	41.11 ± 0.03	102.90 ± 0.07	144.01 ± 0.01
LZ8	+	83.65 ± 0.05	92.81 ± 0.02	176.46 ± 0.05
LZ9	+	4.59 ± 0.02	41.40 ± 0.05	45.99 ± 0.04
LZ222	-	ND	ND	ND

Note: ‘ND’ means not detected.
